# Thyroid nodules: need for a universal risk stratification system

**DOI:** 10.3389/fendo.2023.1209631

**Published:** 2023-07-21

**Authors:** Priyanka Majety

**Affiliations:** Department of Endocrinology, Diabetes and Metabolism, Virginia Commonwealth University Health, Richmond, VA, United States

**Keywords:** thyroid nodule, ultrasound scoring systems, risk stratification, sonographic features, risk calculators

## Introduction

Thyroid nodules are common and are one of the most common reasons for endocrinology clinic encounters. The widespread use of various imaging modalities and improved healthcare access have resulted in a significant increase in the discovery of incidental thyroid nodules. About half of the population develops a thyroid nodule by age 60 that can be found either through physical examination or imaging. Thankfully, 85% to 90% prove benign (1–3). However, in the United States, every year over 500 000 fine-needle aspirations (FNAs) are conducted, with about 200 000 of them being unnecessary. Thus, identifying the nodules at the highest risk of malignancy is critical.

Evaluation of patients with a suspected thyroid nodule must include a thorough medical history and physical examination and a thyroid-stimulating hormone (TSH) level and ultrasound (US) evaluation. The sonographic characteristics of these nodules are used to better assess the risk of malignancy (RoM). Based on large studies, US features that are associated with an increased risk of malignancy (hypoechogenicity, solid composition, microcalcifications/punctate echogenic foci, irregular margins, taller than wide shape) and decreased risk of malignancy (isoechoic nodules, spongiform appearance, simple cystic nodules, comet tail artifacts) have been identified (4–6). No single US feature satisfactorily identifies malignant nodules. Over the years, several risk stratification systems (RSSs) that use a combination of these features to help clinicians identify high-risk nodules have been developed. An ideal RSS would minimize the number of unnecessary FNAs and identify all *clinically significant* thyroid cancers, leading to lower healthcare costs and morbidity.

## Ultrasound scoring systems

Currently available tools to help clinicians risk-stratify thyroid nodules are:

1) Clinical practice guidelines (CPG) from various professional societies,
2) Scoring systems (qualitative or quantitative),
3) Web-based calculators and
4) An interactive algorithm.

In recent years, artificial intelligence (AI) has shown significant promise in the evaluation of thyroid ultrasounds and in stratifying thyroid nodules.

Several professional organizations have developed ultrasound-based RSSs and management guidelines for thyroid nodules, namely, the American College of Radiology Thyroid Imaging Reporting and Data System (ACR TI-RADS), the American Thyroid Association (ATA) guidelines, the European Thyroid Association (ETA, EU-TIRADS), the Korean Society of Thyroid Radiology/Korean Thyroid Association (KSThR/KTA, K-TIRADS), the Chinese Medical Association (C-TIRADS), the American Association of Clinical Endocrinology (AACE), the American College of Endocrinology (ACE), and the Associazione Medici Endocrinologi (AME) (7–13). There are additional RSSs developed by groups of investigators who do not represent professional organizations.

The characteristics of the commonly used RSSs are outlined in **Table 1**. The most commonly used ultrasound RSSs are based on the presence of one or more discrete features with one exception. The ATA system uses discrete features and patterns comprised of a combination of these discrete features.

**Table 1:** Characteristics of major ultrasound risk stratification systems [adapted from reference (14)].

RSS	Classification format	Number of categories	Categories and estimated RoM
2021 AACE/ACE-AME tool/TNAPP	Electronic algorithmic tool that uses history, labs, and combinations of US features	Clinical 2; US features 3	US1 – 1% US2 – 5-15% US3 – 50-90%
2015 ATA	Pattern recognition	5	Benign - <1% Very low - <3% Low - 5–10% Intermediate - 10–20% High - 70–90%
2017 ACR-TIRADS	Point-based system	5	TR1 - <2% TR2 - <2% TR3 - <5% TR4 - 5–20% TR5 - >20%
2017 EU-TIRADS	Algorithmic (combinations of US features)	5	TR1 – None TR2 – 0% TR3 - 2-4% TR4 - 6-17% TR5 - 26-87%
2016 K-TIRADS	Algorithmic (combinations of US features)	5	K-TIRADS 1 – None K-TIRADS 2 – < 3% K-TIRADS 3 – 3-15% K-TIRADS 4 – 15-50% K-TIRADS 5 - > 60%
2020 C-TIRADS	Point-based system	6	C-TR 1 – None C-TR 2 – 0% C-TR 3 – <2% C-TR 4 A– 2-10% C-TR 4 B– 10-50% C-TR 4 C– 50-90% C-TR 5 – >90% C-TR 6 – Proven malignancy

AACE/ACE/AME, American Association of Clinical Endocrinology, American College of Endocrinology, Associazione Medici Endocrinologi; TNAPP, The Thyroid Nodule App; ACR TI-RADS, American College of Radiology Thyroid Imaging Reporting and Data System; ATA, American Thyroid Association; EU-TIRADS, European Thyroid Association Thyroid Imaging Reporting and Data System; K-TIRADS, Korean Society of Thyroid Radiology/Korean Thyroid Association Thyroid Imaging Reporting and Data System; RSS, risk stratification system; RoM, risk of malignancy.

Risk calculators and computer-interpretable guidelines (CIG) are interactive tools where unambiguous, sequential recommendations are made and can be used to engage patients. **Table 2** summarizes the various risk calculators available.

**Table 2:** Summary—thyroid nodule risk calculators [Adapted from reference (14)].

Thyroid nodule risk calculators				
Inputs	Inputs		Outputs	Comments
***ACR TI-RADS & AI TI-RADS:*** ***Websites:*** https://deckard.duhs.duke.edu/~ai-ti-rads/index.html	-Composition -Echogenicity -Shape -Margin -Echogenic foci		- Total points - TI-RADS score - FNA recommendation	Most widely used risk calculator, particularly among radiologists. It is restricted to thyroid US features and size. Clinical factors are not taken into consideration.
***Malignancy risk estimation of lesions with AUS/FLUS:*** ***Website:*** http://www.gap.kr/thyroidnodule_b3.php	- Biopsy results (nuclear vs. architectural atypia) - Diameter - Internal content - Shape - Margin - Echogenicity - Calcification		- Total score - RoM in %	It is restricted to nodules with AUS/FLUS. It provides statistics about the RoM but not guidance about whether to perform FNA.
***The BWH thyroid nodule risk estimator:*** ***Website:*** https://thyroidcancerrisk.brighamandwomens.org/	-Age at time of diagnosis -Sex -Largest diameter -Cystic content -Additional nodules (≥1cm)		RoM in %	***Strengths:*** Simple, reproducible (due to relatively objective data used as inputs), and best suited for populations. ***Weaknesses:*** It is best suited for evaluating RoM in populations rather than individual patients (employs only a limited amount of reproducible data). It provides statistics about the RoM but not guidance about whether to perform FNA.
***The thyroid nodule malignancy risk*** ***calculator - Spain:*** ***Website:*** https://obgynreference.shinyapps.io/calccdt/	Patient characteristics: -Age -Sex -Family history of thyroid cancer (1^st^ degree relatives) -TSH -Autoimmune thyroiditis (positive antibodies)	Nodule characteristics: -Maximum diameter -Content -Echogenicity -Margins -Calcifications -Shape -Suspicious lymph node	- RoM in % - FNA recommendations	Requires data such as anti-thyroid antibodies, which are not routinely performed in the evaluation of thyroid nodules.
***Cleveland Clinic calculator:*** ***Website:*** https://riskcalc.org/ThyroidCancer/	a) FNA – No: -Shape -Vascularity -TSH -Echo texture -Age -Margin -Tumor size -Calcification	b) FNA – Yes: -Shape -Vascularity -TSH -Echo texture -Calcification -Grooves -Pseudo-inclusions -Cellularity -Colloid (Scant or abundant)	RoM in %	Employs vascularity, which is no longer recognized as a key determinant of thyroid malignancy.
***TNAPP:*** ***Website:*** https://aace-thyroid.deontics.com/dwe/int/public/welcome.jsp a) Clinical features b) US features c) Cytology features			-Eligibility for using TNAPP -AACE US category -AACE clinical category -FNA recommendations -ACR TI-RADS risk category -ACR TI-RADS biopsy advice - RoM in % -If FNA available, recommendations on molecular testing, surgery, and follow-up	***Strengths:*** Interactive, comprehensive, paralleling clinical practice guidelines (CPG) guidance. Integrates clinical, sonographic, and cytologic variables together to assess risk. Limited data required for each recommendation. Guides the clinician at various stages: eligibility to use the application, FNA and follow-up advice, and post-FNA advice. Modifies recommendations as more information is provided. ***Weaknesses:*** Requires familiarity with the user interface. Creation of an account login is necessary.

ACR TI-RADS, American College of Radiology Thyroid Imaging Reporting and Data System; RoM, risk of malignancy; AUS: atypia of undetermined significance; FLUS: follicular lesion of undetermined significance; BWH: Brigham and Women’s Hospital; TNAPP, The Thyroid Nodule App.

## Comparison of risk stratification systems

There are considerable differences between the various RSSs. They differ in their formats (pattern recognition versus point systems), risk categories, FNA size thresholds, and in the recommended surveillance intervals (if present). Multiple studies have compared various risk stratification tools, most of them retrospective. No single system has consistently demonstrated superiority over the others (possibly due to differences in the patient populations, inclusion and exclusion criteria, and analytic methods).

A meta-analysis compared five major RSSs, namely, AACE/ACE/AME, ATA, K-TIRADS, ACR TI-RADS, and EU-TIRADS. It included 12 studies with 28,750 nodules (15.2% malignant). In order to avoid the bias arising from the different methodologies of the published studies, summary operating measures that are assumed to be independent of disease prevalence were used, such as the diagnostic odds ratio (DOR). The DOR is the odds of a positive test in those with disease relative to the odds of a positive test in those without disease. The diagnostic odds ratio ranged from 2.2 to 4.9 among the different RSSs. A head-to-head comparison showed a higher relative DOR (RDOR) [1.9, 95% CI (1.3-2.9); P = .002] for ACR-TIRADS [DOR: 5.6, 95% CI (3.4–9.0)] versus ATA [DOR: 2.9, 95% CI (1.3–6.5)] due to a higher relative likelihood ratio for positive results. Similarly, a comparison between ACR-TIRADS [DOR: 4.5, 95% CI (2.5–7.9)] and K-TIRADS [DOR: 2.5 95% CI (1.1-5.6)] showed a higher RDOR [1.8, 95% CI (1.2 – 2.6); P = .002] (15).

Ha et al. studied a total of 2000 consecutive thyroid nodules (≥ 1 cm) in 1802 patients and compared seven society guidelines. Overall, the ACR TI-RADS recommended the fewest “unnecessary” (benign) thyroid nodule FNAs at 25.3%, followed by the 2016 AACE/ACE/AME guidelines (32.5%), ATA (51.7%), and K-TIRADS (56.9%). While the K-TIRADS (94.5%) and ATA (89.6%) guidelines were more sensitive compared with the AACE/ACE/AME (80.4%) and ACR (74.7%), the latter were more specific (ACR 67.3%, AACE/ACE/AME 58%, and ATA 33.2%) (16).

Another meta-analysis compared four RSSs, namely, ACR-TIRADS, EU-TIRADS, ATA, and K-TIRADS. This analysis included 29 different studies with a total of 33,748 nodules with pathological or imaging follow-up. The respective pooled sensitivity and specificity of the various RSSs were:

- ACR-TIRADS: 66% and 91% for category 5 and 95% and 55% for category 4 or 5
- ATA: 74% and 88% for category 5 and 91% and 64% for category 4 or 5
- K-TIRADS: 55% and 95% for category 5 and 89% and 64% for category 4 or 5
- EU-TIRADS: 82% and 90% for category 5 and 96% and 52% for category 4 or 5.

When high-risk categories (categories 4-5) were evaluated, no difference was found between the RSSs (17).

A prospective, observational study from a single thyroid cancer unit of a large hospital analyzed 832 thyroid nodules referred for FNA and compared the performance of five RSSs (ATA, AACE/ACE/AME, ACR TIRADS, EU-TIRADS, and K-TIRADS). All the nodules were classified based on US features and stratified using each of the five RSSs, and the recommendation for FNA was evaluated with the final pathologic diagnosis. After excluding nodules with indeterminate cytology, a total of 502 nodules were included in the final cohort. It was concluded that consistently adhering to any of the RSS guidelines would have reduced the number of FNAs by 17.1% and that ACR-TIRADS allowed the largest reduction (268 of 502) in the number of FNAs with the lowest false-negative rate of 2.2% (95% CI, 95.2% to 99.2%). Although the discriminatory capacities of all the RSSs (except for K-TIRADS) were comparable to that of ACR-TIRADS, they recommended more FNAs (18).

## Discussion

With multiple risk stratification tools available, clinicians choose their tools informed by their geography and specialization. Both these factors select for involvement with particular professional societies, many of which have their own validated risk stratification systems. As discussed above, studies comparing the performance of various RSSs have had inconsistent results. This makes it difficult for clinicians to consistently implement an RSS. The wide variety of systems may often lead to confusion on the part of both patients and physicians due to a lack of uniformity. This is relevant, especially in the era of “open notes”, where patients can access their health records. It can be a puzzling experience when radiologists and clinicians use multiple RSSs with differing management recommendations. It can also be a time-consuming exercise for clinicians to re-evaluate all the nodules using a different RSS, particularly in the fast-paced clinics.

This also poses a challenge to endocrinologists and other clinicians in training. During clinical training, trainees work with several teaching attendings, and many of them have a different approach to thyroid nodule evaluation, the biggest difference being the RSS in use. Some senior clinicians do not use any specific RSS but go with their *intuition*, while others use different RSSs, reflective of the differences in their training and experience. Some radiologists include the ACR-TIRADS classification of nodules in their reports, while others do not. Although this system enables clinicians in training to learn and use one of several RSSs to justify a specific recommendation based on the patient’s medical history, comorbidities, and preferences, it can be an overwhelming and confusing experience.

Another challenge of US-based RSSs is inter- and intra-observer variability (19). When comparing various RSSs, studies have shown that inter-observer agreement is better for intermediate- and high-suspicion nodules than for low-suspicion nodules (20). In another blinded, multi-center study, 100 electronically recorded thyroid nodule US images were analyzed, and the evaluation was repeated four months later after randomization. The analysis was performed by radiologists and endocrinologists. They were also classified according to the ATA, AACE/ACE/AME, EU-TIRADS, and ACR-TIRADS classifications. The aim of this study was to assess inter- and intra-observer agreement between different thyroid centers and different specialists. They concluded that while the intra-observer reproducibility for thyroid nodule US classification appears fairly adequate, the inter-observer agreement between the different centers is lower than in single-center trials (21). There are still inconsistencies in thyroid US examiners’ reporting and rating abilities. A potential solution to this problem is a unified lexicon of thyroid US features and dedicated training. This may increase inter-observer agreement and improve the predictive value of the classification system.

There is a compelling need for a universal risk stratification system that would help not only clinicians but also patients in understanding ultrasound reports and making appropriate recommendations in identifying the nodules that require further evaluation including a biopsy. A grassroots initiative, managed by the steering committee of the International Thyroid Nodule Ultrasound Working Group (ITNUWG), is currently working to develop an international RSS, termed I-TIRADS, that integrates the leading RSSs (22). A recent multidisciplinary international survey conducted by the ITNUWG on RSS-use patterns and practitioner characteristics and preferences confirmed this notion. There were 875 respondents from 52 countries from more than seven specialties. About one-third of the respondents indicated the use of more than one RSS in their practice, potentially leading to confusion, and another third of the respondents reported not using an RSS for various reasons. Most of them supported a comprehensive points-based RSS with no more than five risk categories (23). The majority of them (62% of the respondents) indicated that a universal lexicon paired with illustrative images of ultrasound features would improve inter-observer variability. They also supported the idea of a comprehensive atlas of thyroid US images and videos and dedicated training on the universal lexicon.

There is a strong need for a universal RSS with a lexicon to harmonize all the current systems and standardize the evaluation of thyroid nodules with the aim of reducing unnecessary thyroid biopsies without jeopardizing the detection of clinically significant malignancies. The development of I-TIRADS is a step towards this vision, but we would still need to wait for validation in large population studies.

## Author contributions

The author confirms being the sole contributor of this work and has approved it for publication.
